# Supplementary figures and images for: GMQN: A Reference-Based Method for Correcting Batch Effects and Probe Bias in HumanMethylation BeadChip
Source: Front Genet. 2022 Jan 7;12:810985. doi: 10.3389/fgene.2021.810985 (PMC8777061; doi:10.3389/fgene.2021.810985)

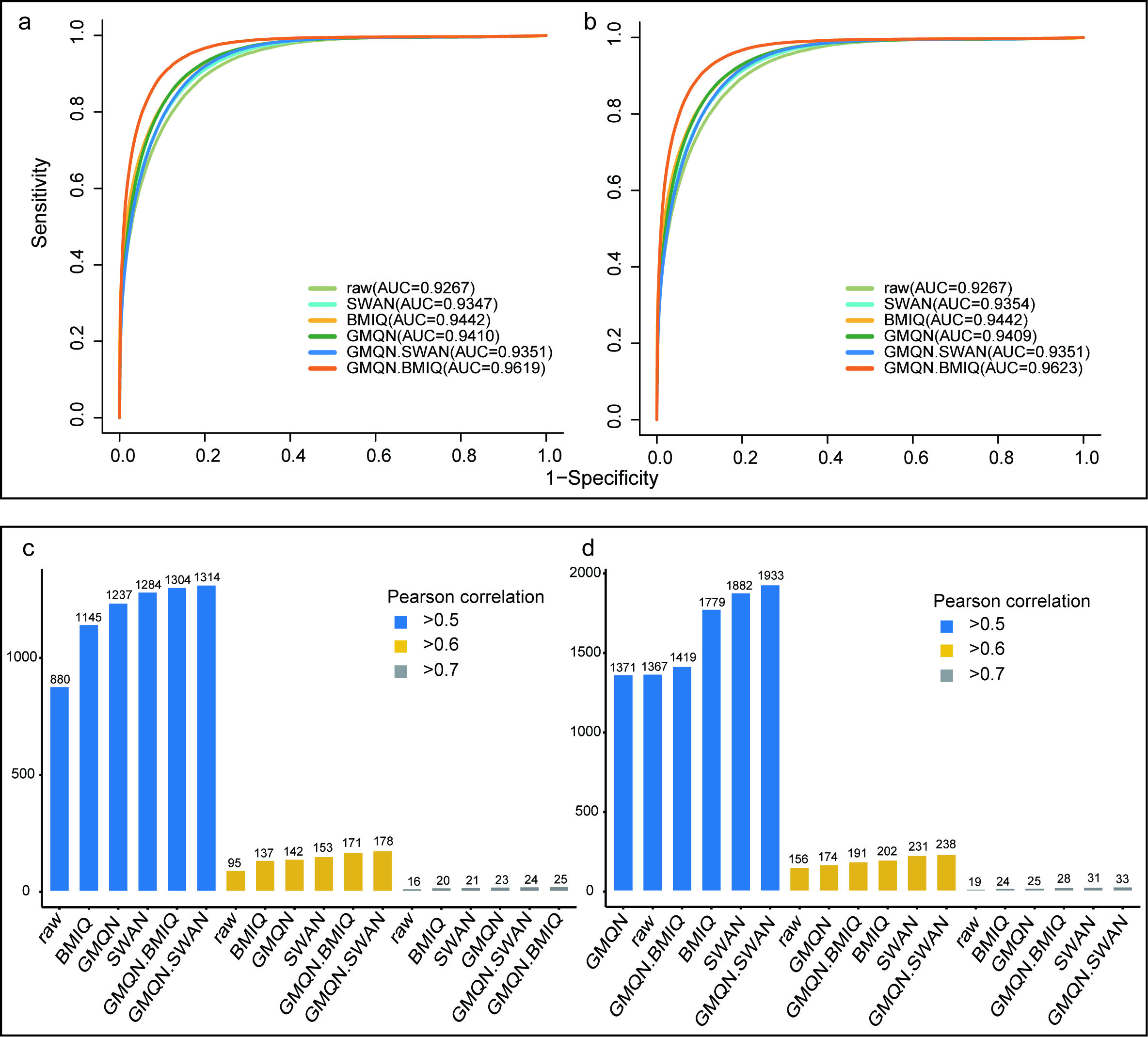

Supplement: Supplementary file 1 [file Image2.TIF]

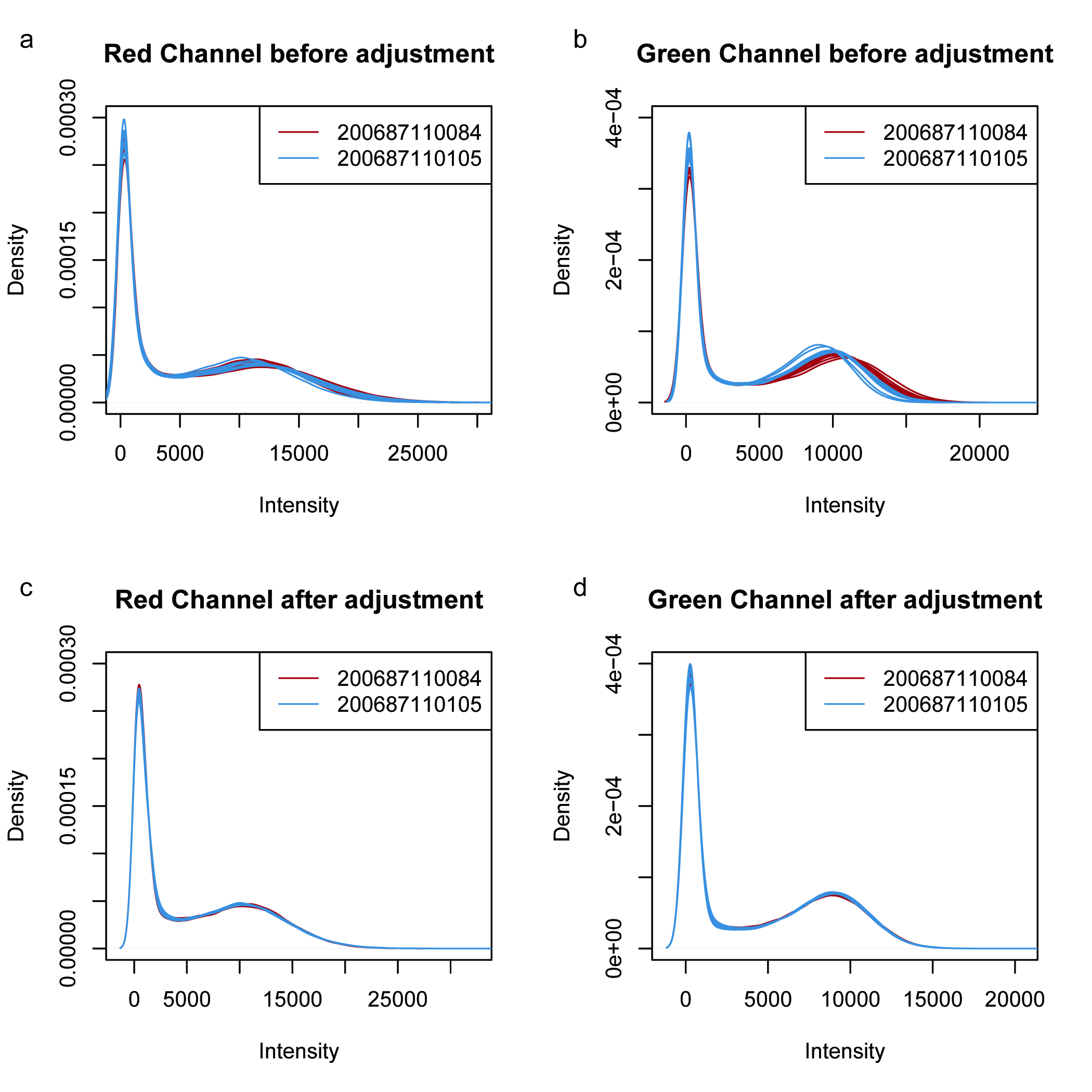

Supplement: Supplementary file 2 [file Image1.TIF]
